# Enlargement of Ribbons in Zebrafish Hair Cells Increases Calcium Currents But Disrupts Afferent Spontaneous Activity and Timing of Stimulus Onset

**DOI:** 10.1523/JNEUROSCI.2878-16.2017

**Published:** 2017-06-28

**Authors:** Lavinia Sheets, Xinyi J. He, Jennifer Olt, Mary Schreck, Ronald S. Petralia, Ya-Xian Wang, Qiuxiang Zhang, Alisha Beirl, Teresa Nicolson, Walter Marcotti, Josef G. Trapani, Katie S. Kindt

**Affiliations:** ^1^Section on Sensory Cell Development and Function, National Institute on Deafness and Other Communication Disorders/National Institutes of Health, Bethesda, Maryland 20892,; ^2^Department of Otolaryngology, Harvard Medical School, Boston, Massachusetts 02115,; ^3^Eaton-Peabody Laboratory, Massachusetts Eye and Ear, Boston, Massachusetts 02114,; ^4^Department of Biology and Neuroscience Program, Amherst College, Amherst, Massachusetts 01002,; ^5^Oregon Hearing Research Center and Vollum Institute, Oregon Health and Science University, Portland, Oregon 97239,; ^6^Department of Biomedical Science, University of Sheffield, Sheffield S10 2TN, United Kingdom, and; ^7^Advanced Imaging Core, National Institute on Deafness and Other Communication Disorders/National Institutes of Health, Bethesda, Maryland 20892

**Keywords:** calcium channels, hair cell, ribbon synapse, sensory, synapse, zebrafish

## Abstract

In sensory hair cells of auditory and vestibular organs, the ribbon synapse is required for the precise encoding of a wide range of complex stimuli. Hair cells have a unique presynaptic structure, the synaptic ribbon, which organizes both synaptic vesicles and calcium channels at the active zone. Previous work has shown that hair-cell ribbon size is correlated with differences in postsynaptic activity. However, additional variability in postsynapse size presents a challenge to determining the specific role of ribbon size in sensory encoding. To selectively assess the impact of ribbon size on synapse function, we examined hair cells in transgenic zebrafish that have enlarged ribbons, without postsynaptic alterations. Morphologically, we found that enlarged ribbons had more associated vesicles and reduced presynaptic calcium-channel clustering. Functionally, hair cells with enlarged ribbons had larger global and ribbon-localized calcium currents. Afferent neuron recordings revealed that hair cells with enlarged ribbons resulted in reduced spontaneous spike rates. Additionally, despite larger presynaptic calcium signals, we observed fewer evoked spikes with longer latencies from stimulus onset. Together, our work indicates that hair-cell ribbon size influences the spontaneous spiking and the precise encoding of stimulus onset in afferent neurons.

**SIGNIFICANCE STATEMENT** Numerous studies support that hair-cell ribbon size corresponds with functional sensitivity differences in afferent neurons and, in the case of inner hair cells of the cochlea, vulnerability to damage from noise trauma. Yet it is unclear whether ribbon size directly influences sensory encoding. Our study reveals that ribbon enlargement results in increased ribbon-localized calcium signals, yet reduces afferent spontaneous activity and disrupts the timing of stimulus onset, a distinct aspect of auditory and vestibular encoding. These observations suggest that varying ribbon size alone can influence sensory encoding, and give further insight into how hair cells transduce signals that cover a wide dynamic range of stimuli.

## Introduction

Hair cells, the sensory receptors of auditory, vestibular, and lateral-line organs, use specialized ribbon synapses to encode the timing and intensity of sensory information. Hair-cell ribbon synapses are capable of rapid neurotransmitter release within milliseconds of stimulus onset, and sustained neurotransmitter release over many seconds and longer (Parsons et al., 1994; Moser and Beutner, 2000; Matthews and Fuchs, 2010). What features enable the hair-cell ribbon synapse to perform such fine tasks is not well understood.

Hair-cell ribbon synapses are defined by a unique presynaptic structure known as the synaptic ribbon, which is a dense specialization that tethers glutamate-filled vesicles adjacent to clusters of the presynaptic calcium channel Ca_V_1.3 (Usukura and Yamada, 1987; Brandt et al., 2005; Obholzer et al., 2008; Seal et al., 2008; Schmitz, 2009; Frank et al., 2010). The major component of ribbons is Ribeye, a unique protein that is vital for the physical integrity and function of ribbon synapses (Schmitz et al., 2000; Zenisek et al., 2004; Frank et al., 2010; Sheets et al., 2011; Lv et al., 2016). The size and shape of ribbons vary depending on species and hair-cell type, but it is unclear how these differences impact synapse function (Moser et al., 2006). Ribbons have been shown to tether and stabilize vesicles at the presynaptic active zone (Smith and Sjostrand, 1961; Khimich et al., 2005; Buran et al., 2010). The number of tethered vesicles increases with larger ribbons (for review, see Nouvian et al., 2006), although the functional implications of the additional vesicles are not clear. Previous studies have also shown that hair-cell ribbons are able to recruit Ca_V_1.3 channels, indicating an intimate relationship between these structures (Frank et al., 2010; Sheets et al., 2011, 2012; Wong et al., 2014; Lv et al., 2016).

In the mammalian auditory system, substantial work has been done in auditory inner hair cells (IHCs) to determine how ribbon size, Ca_V_1.3 channels, and vesicle populations ultimately impact sensory encoding (Pfeiffer and Kiang, 1965; Liberman, 1982; Taberner and Liberman, 2005; Johnson et al., 2008). IHCs are innervated by multiple afferent-nerve fibers, and there is evidence that synapses with larger ribbons and smaller postsynapses have a low rate of spontaneous release and correspond to high-threshold nerve fibers, while synapses with smaller ribbons and larger postsynapses show a higher rate of spontaneous release and correspond to low-threshold nerve fibers (Liberman et al., 2011). Whether differences in afferent activity are due to morphological differences at the postsynapse or at the ribbon is not well understood. In auditory IHCs, larger ribbons have also been shown to localize more Ca_V_1.3 channels and have larger synaptic calcium signals compared with smaller ribbons (Meyer et al., 2009; Ohn et al., 2016). Despite larger calcium signals, larger ribbons were not correlated with more afferent activity, which has been attributed to a depolarizing shift in calcium channel activation present at larger ribbons compared with smaller ribbons. (Ohn et al., 2016). Overall, additional differences in Ca_V_1.3 channel numbers and variability in the size of the postsynapse across ribbons in IHCs make it is difficult to isolate the impact of ribbon size on auditory encoding.

To address how ribbon size influences hair-cell ribbon-synapse function, we used a transgenic zebrafish line that overexpresses Ribeye and enlarges ribbons (Sheets et al., 2011). Although hair-cell ribbons are enlarged in this transgenic line, there was no significant effect on postsynaptic size. Using a multipronged approach, we used this transgenic line as a model to understand how hair-cell ribbon size alters ribbon-synapse morphology and function. We found, at the ultrastructural level, that enlarged ribbons have more associated synaptic vesicles yet a similar number of docked vesicles. Functionally, both global and ribbon-localized calcium signals are increased in hair cells with enlarged ribbons. Despite increased calcium signaling, presynaptic Ca_V_1.3 channel density does not appear to scale up with ribbon enlargement, and channel density is reduced. Despite increases in calcium current and more associated vesicles, ribbon enlargement resulted in a reduction in spontaneous action potentials, and a longer latency to fire following stimulus onset.

## Materials and Methods

### 

#### 

##### Fish strains and reagents.

Adult zebrafish (*Danio rerio*) were maintained with a 14 h light, 10 h dark cycle using standard methods. Zebrafish work performed at the National Institutes of Health was approved by the Animal Use Committee at the National Institutes of Health (animal study protocol #1362-13). At Oregon Health and Sciences University, zebrafish work was overseen by the Institutional Animal Care and Use Committee. At Massachusetts Eye and Ear, zebrafish work was performed with the approval of the Massachusetts Eye and Ear Animal Care Committee and in accordance with National Institutes of Health guidelines for use of zebrafish (protocol #13-001A). All zebrafish work at the University of Sheffield was licensed by the United Kingdom Home Office under the Animals (Scientific Procedures) Act 1986 and approved by the University of Sheffield Ethical Review Committee. Larvae were examined at 3–7 d post fertilization (dpf) unless stated otherwise. At these ages, sex cannot be predicted or determined; therefore, sex of the animal was not considered in our studies. Zebrafish larvae were raised in E3 embryo media in mm as follows: 5 NaCl, 0.17 KCl, 0.33 CaCl_2_, and 0.33 MgSO_4_, buffered in HEPES, at 30°C. All wild-type (WT) controls were nontransgenic siblings unless stated otherwise. Previously described transgenic zebrafish strains used in this study include the following: *Tg(-6myo6b:ribeye b-EGFP)^vo67Tg^, Tg(-6myo6b:RGECO)^vo10Tg^* and *Tg(-6myo6b:GCaMP6s-CAAX)^idc1Tg^* (Sheets et al., 2011; Maeda et al., 2014; Jiang et al., 2017).

##### Vector construction and transgenic lines.

To create additional Ribeye transgenic fish, plasmid construction was based on the tol2/Gateway zebrafish kit developed by the lab of Chi-Bin Chien at the University of Utah (Kwan et al., 2007). *Ribeye a* (NCBI Accession Number NM_001195491.1) and *ribeye b* (NM_001015064.1) were cloned into the middle entry vector pDONR221 to create pME-*ribeye a* or pME-*ribeye b*. From the tol2 kit, vectors p3E-*mCherry* (388), pDestTol2 (395, 394), and p3E-polyA (302) were recombined with p5E-*6myosin6b* (Kindt et al., 2012) and our engineered plasmids to create the following constructs: -*6myosin6b:ribeye a-mCherry*, and *-6myosin6b:ribeye b-mCherry.*

To generate transgenic fish from these constructs, plasmid DNA (25–50 ng/μl), along with *tol2* transposase mRNA (25–50 ng/μl), was injected into zebrafish embryos at the single-cell stage. Transgenic lines were screened in the F1 and F2 generation for single-copy integrations and expression level. The *Tg(-6myo6b:ribeye b-mCherry)^idc3Tg^* transgenic strain was selected because, using immunolabel (see methods below), it had normal number and size of ribbons compared with WT (ribbon area normalized to the WT median area, WT: 0.924 ± 0.073 a.u., *n* = 245 ribbons; *ribeye b-mCherry*: 0.909 ± 0.051 a.u., *n* = 264 ribbons, *p* = 0.867; synapses per hair cell via immunolabel: WT: 3.06 ± 0.13, *n* = 8 neuromasts; *ribeye b-mCherry*: 2.97 ± 0.14, *n* = 6 neuromasts, *p* = 0.601). *Tg(-6myo6b:ribeye a-mCherry)^idc2Tg^* was chosen because, similar to the *Tg(-6myo6b:ribeye b-EGFP) ^vo67Tg^* transgenic strain, two copies of *Tg(-6myo6b:ribeye a-mCherry)^idc2Tg^* resulted in ribbons that were significantly enlarged compared with WT (ribbon area normalized to the WT median area, WT: 0.924 ± 0.073 a.u., *n* = 245 ribbons; *ribeye a-mCherry* × *2*: 1.90 ± 0.190 a.u., *n* = 377 ribbons, *p* = 0.0006; synapses per hair cell via immunolabel: WT: 3.06 ± 0.13 *n* = 8 neuromasts; *ribeye a-mCherry* × *2*: 2.86 ± 0.14, *n* = 8 neuromasts, *p* = 0.304). This analysis was performed on *z*-stack images acquired on a LSM780 microscope (Carl Zeiss; see methods below). All Ribeye transgenic fish used in this study had a similar number of hair cells, and a normal startle reflex and balance, indicating that our transgenes do not overtly alter auditory or vestibular function.

For electron microscopy, immunohistochemistry, whole-cell recordings, and afferent recordings, an in-cross of *Tg(-6myo6b:ribeye b-EGFP)^vo67Tg^* was used to compare larvae with 2 copies of Ribeye b-EGFP to WT, nontransgenic siblings. For cytosolic calcium measurements, *Tg(-6myo6b:RGECO1)^vo10Tg^; Tg(-6myo6b:ribeye b-EGFP)^vo67Tg^ × 2* triple transgenic hair cells were compared with *Tg(-6myo6b:RGECO1)^vo10Tg^* single transgenic hair cells. For ribbon-localized calcium responses, *Tg(-6myo6b:GCaMP6s-CAAX)^idc1Tg^; Tg(-6myo6b:ribeye a-mCherry)^idc2Tg^ × 2* triple transgenic hair cells with enlarged ribbons were compared with *Tg(-6myo6b: GCaMP6s-CAAX)^idc1Tg^*; *Tg(-6myo6b:ribeye b-mCherry)^idc3Tg^* double-transgenic hair cells with WT-sized ribbons.

##### Zebrafish immobilization and hair cell mechanical stimulation.

To suppress muscle activity, larvae were anesthetized with 0.03% 3-amino benzoic acid ethyl ester (MS-222, Western Chemical), mounted with tungsten pins, and microinjected in the heart with 125 μm α-bungarotoxin (Tocris Bioscience) to suppress muscle activity. Larvae were then rinsed and maintained in normal extracellular solution in mm as follows: 130 NaCl, 2 KCl, 2 CaCl_2_, 1 MgCl_2_, and 10 HEPES, pH 7.3, 290 mOsm. Stimulation of neuromast hair cells was performed as described previously (Trapani and Nicolson, 2010). Briefly, we used a pressure clamp (HSPC-1, ALA Scientific) attached to a glass micropipette (inner tip diameter ∼ 30 μm) filled with normal extracellular solution to mechanically stimulate hair cells. The waterjet pipette was positioned (MP-265, Sutter Instruments) ∼100 μm from a given neuromast and displacement (3–5 μm) of the kinocilial tips was verified by eye. For recordings of lateral-line afferents, the pressure clamp was driven by a voltage command delivered by the recording amplifier and pressure was monitored from a feedback sensor located on the HSPC-1 headstage and collected concurrently. For calcium imaging experiments, the pressure clamp was driven by a voltage step command. An outgoing voltage signal from the imaging software was used to coordinate imaging with the pressure clamp stimulus.

##### Electrophysiology, lateral-line afferent recordings.

Our recording setup for action currents has been described in detail previously (Trapani and Nicolson, 2010; Olt et al., 2016b). For all experiments, recordings were performed in normal extracellular solution (see above) on afferent neurons innervating zebrafish primary neuromasts (L1-L4). For extracellular recordings, borosilicate glass pipettes were pulled (P-97, Sutter Instruments) with a long taper and had resistances between 5 and 15 mΩ in extracellular solution. Signals were collected with a Multiclamp 700B, a Digidata 1550 data acquisition board, along with pClamp10 software (Molecular Devices). Extracellular currents were acquired from an individual lateral-line afferent neuron in the loose-patch configuration (seal resistances ranged from 20 to 80 mΩ in extracellular solution). Recordings were done in voltage-clamp mode, sampled at 50 μs/data point, and filtered at 1 kHz. Spontaneous spike rate was quantified from measurements of 500 spontaneous events per neuron. The innervated neuromast for a recorded neuron was identified by progressively stimulating primary neuromasts of the posterior lateral line until phase-locked spiking was detected.

##### Electrophysiology, lateral-line hair-cell recordings.

Whole-cell patch clamp experiments were performed from hair cells of the zebrafish primary neuromasts (L1-L4) as previously described (Olt et al., 2014, 2016b). The zebrafish were placed in a microscope chamber, with continuous perfusion via a peristaltic pump in the following extracellular solution in mm: 135 NaCl, 1.3 CaCl_2_, 5.8 KCl, 0.9 MgCl_2_, 0.7 NaH_2_PO_4_, 5.6 d-glucose, 10 HEPES-NaOH. Sodium pyruvate (2 mm), MEM amino acids solution (50×, without l-glutamine), and MEM vitamins solution (100×) were added from concentrates (Fisher Scientific). The pH was 7.5. For calcium current recordings, the extracellular solution was as the above but with 2.8 mm CaCl_2_ instead of 1.3 mm (NaCl was reduced to 133 mm to keep the osmolality of the solution constant).

Calcium current and changes in membrane capacitance recordings were conducted at zebrafish body temperature (28.5°C). All other experiments (examination of K^+^ currents and voltage responses) were performed at room temperature (21°C–24°C). Patch pipettes were made from soda glass capillaries (Harvard Apparatus) and had a typical resistance in the extracellular solution of 3–5 mΩ. To reduce the fast electrode capacitive transient, the shank of each capillary was coated with surfboard wax (Mr. Zog's SexWax, Sexwax). Current and voltage recordings were performed using the following intracellular solution in mm: 131 KCl, 3 MgCl_2_, 1 EGTA-KOH, 5 Na_2_ATP, 5 HEPES-KOH, and 10 sodium phosphocreatine, pH 7.3. For calcium current recordings and capacitance measurements, the intracellular solution contained the following in mm: 85 Cs-glutamate, 20 CsCl, 3 MgCl_2_, 1 EGTA-CsOH, 5 Na_2_ATP, 5 HEPES-CsOH, 10 Na_2_-phosphocreatine, 0.3 Na_2_GTP, 15 4-aminopyridine, and 20 tetraethyl ammonium, pH 7.3. Recordings were made with an Optopatch amplifier (Cairn Research). Data acquisition was performed using pClamp software with a Digidata 1322A data acquisition board (Molecular Devices). Recordings were sampled at 5 or 100 kHz, low pass filtered at 2.5 or 10 kHz (8-pole Bessel) and stored on computer for offline analysis using Origin 2016 (OriginLab) and pClamp 10 (Molecular Devices). Membrane potentials in voltage clamp were corrected for the voltage drop across the uncompensated residual series resistance (*R*s: 5.3 ± 0.5 mΩ, *n* = 56) and for a liquid junction potential, measured between electrode and bath solutions, of −4 mV for the KCl-based and −9 mV for Cs-glutamate-based intracellular solution. Current responses are referred to a holding potential of −84 mV or −79 mV, and are set to 0-current for easy comparison between recordings from different hair cells.

##### Calcium imaging.

Optical measurements were made as previously described (Kindt et al., 2012; Zhang et al., 2016). Briefly, calcium imaging experiments were performed in normal extracellular solution (see above). Recordings were made at 10 Hz for cytosolic RGECO1 measurements and 20 Hz for ribbon-localized GCaMP6s-CAAX measurements. For cytosolic calcium measurements using RGECO1, a Nikon Eclipse NiE widefield system with a 60× 1.0 NA CFI Fluor water-immersion objective was used with excitation: 540/25 565LP and emission: 620/60 filters. The microscope was equipped with an Orca D2 camera (Hamamatsu), controlled using Elements software (Nikon Instruments). For cytosolic RGECO1 measurements, a central imaging plane at the level of hair cell nucleus was used. Calcium measurements at presynaptic ribbons made using GCaMP6s-CAAX were acquired on a Swept-field confocal system built on a Nikon FN1 upright microscope (Bruker) with a 60× 1.0 NA CFI Fluor water-immersion objective. The microscope was equipped with a Rolera EM-C2 EMCCD camera (QImaging), controlled using Prairie view (Bruker). GCaMP6s-CAAX and Ribeye-mCherry were excited using 488 and 561 nm solid state lasers. The microscope was equipped with a Dual-View beam splitter (Photometrics) using the following filters: dichroic 565; GCaMP6 emission 520/30; mCherry emission 630/50 (Chroma) to enable dual imaging of GCaMP6s-CAAX calcium signals and Ribeye-mCherry to detect ribbon location. The Ribeye-mCherry signal was used to select a GCaMP6s-CAAX imaging plane containing ribbons in multiple hair cells. For comparisons, calcium imaging experiments were done using a minimum of 4 animals and 8 neuromasts per group.

The L-type calcium channel antagonist isradipine (Sigma-Aldrich) was prepared in normal extracellular solution with 0.1% DMSO and used at 10 μm. Larvae were incubated in drugs for 10 min before calcium imaging.

##### Transmission electron microscopy (TEM).

For electron microscopy, 4 dpf WT siblings and *ribeye b-EGFP* transgenic larvae were fixed in freshly prepared 2% PFA and 4% glutaraldehyde (Electron Microscopy Sciences) in 0.1 m phosphate buffer, pH 7.4, for 30 min at room temperature, followed by a 2 h incubation at 4°C. Larvae were washed with 0.1 m cacodylate buffer 3 × 5 min, and then fixed in 2% glutaraldehyde for 15 min, and washed again with 0.1 m cacodylate buffer 3 × 5 min. Larvae were then placed in 0.1 m osmium tetroxide buffer for 30 min and then washed with 0.1 m cacodylate buffer 3 × 10 min. Larvae were then dehydrated in ethanol: 3 × 5 min 50% ethanol, 15 min 50% ethanol with 1% uranyl acetate, then 2 × 5 min 75% ethanol, 1 × 10 min 95% ethanol, 3 × 10 min 100% ethanol. After dehydration, larvae were placed in propylene oxide (PO), and the incubated in Epon:PO:: 1:1 for 1 h, Epon:PO:: 2:1 for 1 h, and last pure Epon overnight. Epon-embedded samples were then placed in an oven at 64°C for 24 h. Transverse serial sections (60–80 nm thin sections) were placed on a single-slot, formvar/carbon coated nickel grid (2 × 1 mm, Electron Microscopy Sciences) and used to section through cranial neuromasts located between the eyes, or neuromasts located between the eye and the ear. Samples were imaged on a JEM-2100 electron microscope (JEOL). Whenever possible, serial sections were used to restrict our analysis to central sections of ribbons that were directly adjacent to a plasma membrane and near a well-defined afferent postsynaptic density. For our analysis, we analyzed 6 *ribeye b-EGFP* and 9 WT neuromasts, examining up to 7 sections per neuromast. Micrographs containing ribbons were scored blinded. Vesicles with a diameter of 30–50 nm and adjacent (within 60 nm of the ribbon) to the filamentous “halo” surrounding the ribbon were counted as tethered vesicles. Readily releasable vesicles were defined as vesicles between the ribbon and the plasma membrane. The distance of tethered vesicles from the ribbon was defined as the linear distance between the edge of a vesicle and the point on the ribbon closest to it. Average distance was calculated from five independent measurements of tethered, but not readily releasable, vesicles around the perimeter of a ribbon. All distances and perimeters were measured in ImageJ (Schneider et al., 2012).

##### Whole-mount immunohistochemistry.

Zebrafish larvae were fixed with 4% PFA and 4% sucrose in phosphate buffer with 0.2 mm CaCl_2_ for 4.5–6 h at 4°C. Larvae were then permeabilized with ice-cold acetone for 5 min and blocked with PBS buffer containing 2% goat serum, 1% BSA, and 1% DMSO. Primary antibodies were diluted in PBS buffer containing 1% BSA and 1% DMSO, and larvae were incubated in the solution overnight at 4°C. Custom-made primary antibodies for Ribeye a (rabbit polyclonal, 1:500), Ribeye b (IgG2a, 1:2000), and Ca_V_1.3a (rabbit polyclonal, 1:1000), and a commercially available antibody for membrane-associated guanylate kinases (MAGUKs, IgG1, 1:500, NeuroMab AB_10698179) have been described and used previously (Sheets et al., 2011). After removal of primary antibodies, diluted secondary antibodies coupled to Alexa-488, Alexa-647 (Invitrogen), or DyLight 549 (Jackson ImmunoResearch Laboratories) were added. Hair-cell nuclei were labeled with DAPI (Invitrogen).

##### Confocal imaging.

**Figure 1.**
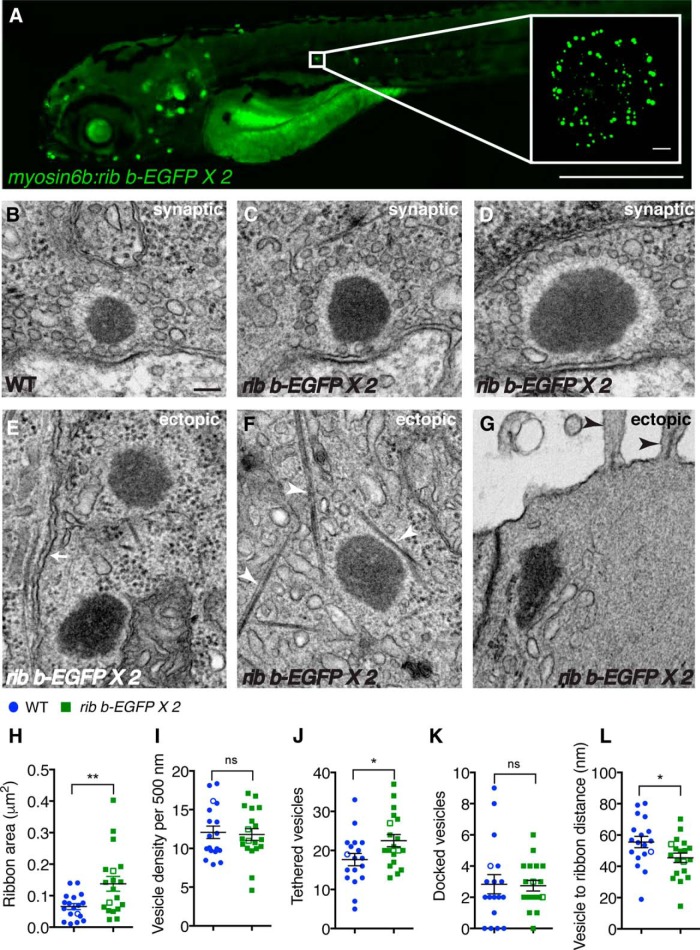
Overexpression of Ribeye b-EGFP increases ribbon size and number of tethered vesicles. ***A***, Image of a live transgenic zebrafish expressing two copies of *ribeye b-EGFP* (*rib b-EGFP* × *2*) in hair cells at 4 dpf. Inset, Top-down projection of a neuromast cluster of lateral-line hair cells expressing Ribeye b-EGFP. ***B–D***, TEM images of neuromast ribbons in WT siblings (***B***) and *ribeye b-EGFP* transgenic fish (***C***, ***D***) at 4 dpf. ***E–G***, Ectopic ribbons in hair cells overexpressing Ribeye b-EGFP. Shown are ectopic ribbons located above the nucleus. Ectopic ribbons are located by the plasma membrane (***E***), associated with filaments (***F***), and near the cuticular plate (***G***). ***E***, White arrow indicates the plasma membrane of the hair cell. ***F***, White arrowheads indicate filamentous structures in the cytosol. ***G***, Black arrowheads indicate stereocilia at the apex of the hair cell. ***H–L***, Quantification of TEM images from *n* = 18 WT and *n* = 19 *ribeye b-EGFP* ribbons from 9 and 6 neuromasts, respectively. In *ribeye b-EGFP* hair cells, ribbon area (***H***) and number of tethered vesicles (***J***) were increased, whereas vesicle density (***I***) and number of docked vesicles (***K***) were not altered. Compared with WT, vesicles were slightly closer to the ribbon in *ribeye b-EGFP* hair cells (***L***). *p < 0.05 (*t* test). **p < 0.01 (*t* test). Scale bars: ***A***, 500 μm; Inset, 5 μm; ***B–G***, 100 nm. ***H–L***, Open circles or squares represent the example images shown in ***B–D***.

Confocal images of fixed samples were obtained as previously described (Sheets et al., 2011). Briefly, *z*-stack images of whole neuromasts (spaced by 0.3 μm over 5–10 μm) were acquired with an Olympus FV1000, a Leica SP8, or a Zeiss LSM780 confocal microscope using 60× 1.3 NA oil, 63× 1.3 NA glycerol, or 63× 1.4 NA oil-immersion objectives, respectively. For quantitative measurements, confocal imaging parameters, including gain, laser power, scan speed, dwell time, resolution, and zoom, were maintained between comparisons. For super resolution imaging (see Fig. 3*F*,*G*), a Zeiss LSM780 microscope with Airyscan was used with a 63× 1.4 NA oil objective, a digital zoom of 25×, acquired at 256 × 256 in 0.19 μm sections. Images were processed in Zen (Carl Zeiss) with an Airyscan processing factor of 6.0. For live images of samples (see Figs. 1*A*, 5*A*,*B*, 6*A*,*B*), a Nikon C2 (10× 0.3 NA air or 60× 1.0 NA water objective) confocal system was used to image and excite EGFP, GCaMP6s, RGECO, or mCherry using the appropriate solid-state laser. For each experiment, the microscope parameters were adjusted using the brightest control specimen.

##### Confocal image processing.

Maximal projections of *z*-stack confocal images were created and analyzed using MetaMorph (Molecular Devices) or ImageJ software. Images containing immunolabel were corrected for background; within maximum-intensity projections, a 7 μm^2^ region containing the highest level of background was selected, and the average-fluorescence intensity of that region was subtracted from each pixel within the image stacks.

To quantitatively measure fluorescent intensities and areas of immunolabeled puncta in MetaMorph, individual neuromasts were delineated using the region tool, and between comparisons, the same inclusive threshold was applied to isolate the pixels occupied by immunolabeled puncta within the neuromast. A punctum was defined as a region of immunolabel where the pixel intensity was at least threefold (Ribeye) or fivefold (Ca_V_1.3a and MAGUK) above the average intensity measured in the whole neuromast. Once the appropriate threshold was applied, the Integrated Morphometry Analysis function was used to automatically quantify the number of puncta, the area of each immunolabeled punctum, and the integrated intensity of fluorescent pixels within each individual punctum.

**Figure 2.**
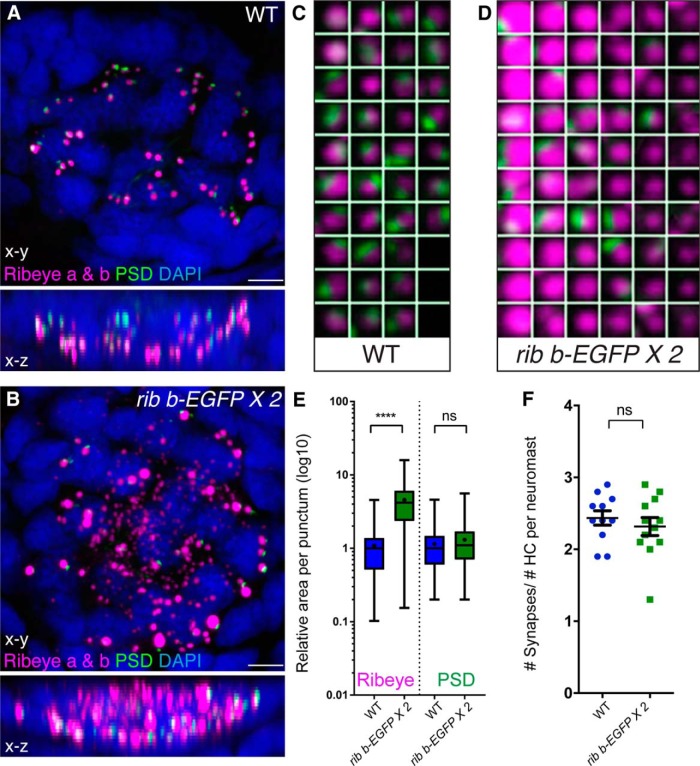
Overexpression of Ribeye b-EGFP increases the size of ribbons but does not increase the size or number of PSDs. ***A***, ***B***, Representative maximum intensity top-down (*x-y*) and side-view (*x-z*) projections of WT (***A***) and transgenic *ribeye b-EGFP* (***B***) neuromasts with Ribeye a and Ribeye b labeling ribbons (magenta) and MAGUK labeling PSDs (green) at 5 dpf. Ribeye a and Ribeye b were labeled with two spectrally separate fluorophores but were both merged as magenta in the displayed images. Cell nuclei were labeled with DAPI (blue). ***C***, ***D***, High-power confocal 1 μm^2^ thumbnails of synapses sorted by size in a representative WT (***C***) and a *ribeye b-EGFP* neuromast (***D***). ***D***, A number of ribbons do not have corresponding PSDs (green). ***E***, Relative area (normalized to WT median) of Ribeye puncta adjacent to PSDs (*n* = 192 WT and *n* = 132 *ribeye b-EGFP*) and MAGUK puncta at ribbon-localized PSDs (*n* = 192 WT and *n* = 210 *ribeye b-EGFP* at 5 dpf). ***F***, Number of complete synapses per hair cell (HC), estimated by taking the total number of synapses per neuromast, divided by the number of hair cells per neuromast (*n* = 11 WT and *n* = 12 *ribeye b-EGFP* neuromasts). Each circle represents a single neuromast in an individual zebrafish. Scale bars: ***A***, ***B***, 5 μm. ****p < 0.0001 (Mann–Whitney *U* test). Synapse counts were not significantly different (unpaired *t* test).

Ca_V_1.3a-immunolabeled puncta adjacent or juxtaposing MAGUK immunolabel (i.e., Ca_V_1.3a puncta that, due to the resolution limits of light microscopy, appeared to partially overlap with MAGUK immunolabel) (Sheets et al., 2012) were defined as presynaptic. To determine whether ribbons were adjacent to postsynaptic densities (PSDs), custom software written in C2+ was used as previously described (Liberman et al., 2011) to produce, for each ribbon or PSD, a thumbnail image of an *x-y* maximum projection of a voxel cube (1 μm^2^) extracted from confocal image stacks and centered on each ribbon synapse using independently derived *x*, *y*, and *z* coordinates of all ribbons and PSDs. The numbers of intact ribbon synapses (i.e., ribbons adjacent to PSDs) were determined by visual inspection of these thumbnail arrays (see Fig. 2*C*,*D*). Subsequent image processing for display within figures was performed using Photoshop and Illustrator software (Adobe).

##### Signal analysis and statistics.

Afferent electrophysiology data were analyzed using custom software written in Igor Pro (Wavemetrics) and were plotted with Igor Pro and Prism 7 (GraphPad). For calcium imaging, image registration and peak detection were performed using custom scripts written in MATLAB (The MathWorks) as described previously (Hilliard et al., 2005; Zhang et al., 2016). For RGECO1 calcium measurements, a circular region of interest (ROI) with a diameter of 3 μm (215 nm per pixel) was placed on each hair cell within a neuromast. For ribbon-localized GCaMP6s-CAAX measurements, a circular ROI with a 1 μm diameter (268 nm per pixel) was placed on the center of an individual ribbon. Ribbon location was determined by either simultaneous or subsequent image capture of Ribeye-mCherry labeled ribbons.

Values in the text and data on graphs are expressed as mean ± SEM. Whenever possible, an effort was made to minimize Type II error with appropriate population numbers. In some of our more challenging electrophysiology experiments with low *N* values, we were not able to achieve a power of >0.8 to indicate no significant difference. In these cases, we state, “we were unable to detect differences” between the two populations. Where appropriate, datasets were confirmed for normality using a Kolmogorov–Smirnov normality test, and for equal variances using a *F* test to compare variances. Statistical significance between two conditions was determined by either paired or unpaired, two-tailed Student's *t* tests, or a Mann–Whitney *U* test, as appropriate. When multiple *t* tests were performed on the same data, a Holm–Sidak method was used to correct for multiple comparisons.

## Results

To examine how ribbon enlargement altered ribbon-synapse morphology and synapse activity in hair cells, we took advantage of a transgenic zebrafish line with enlarged ribbons. In a previous study, we created a stable transgenic line *Tg[myosin6b:ribeye b-EGFP]* that overexpresses exogenous Ribeye b fused to EGFP in zebrafish inner ear hair cells and in neuromast hair cells in the lateral-line system. Upon in-crossing this transgenic strain, we observed high levels of Ribeye b expression in hair cells (Fig. 1*A*; expressing 2 copies and subsequently referred to as *ribeye b-EGFP*) and substantially enlarged ribbons (Sheets et al., 2011).

### Ribeye b-EGFP-expressing hair cells have larger ribbons and more tethered synaptic vesicles

Before investigating synaptic activity, we characterized the synaptic architecture of hair cells with enlarged synapses and compared these measurements with WT synapses. We examined TEM sections that featured ribbon bodies adjacent to postsynaptic densities in zebrafish neuromasts from WT (*n* = 18) and *ribeye b-EGFP* transgenic (*n* = 19) ribbons. We used TEM to quantify ribbon-body areas and found that ribbon areas were significantly larger (2×) in *ribeye b-EGFP* hair cells compared with WT hair cells (Fig. 1*B–D*,*H*; ribbon area, WT: 0.065 ± 0.009 μm^2^*; ribeye b-EGFP*: 0.138 ± 0.023 μm^2^, *p* = 0.008).

We predicted that enlargement of ribbons would increase the number and distribution of synaptic vesicles associated with the ribbon. Synaptic vesicles were defined as circular structures 30–50 nm in diameter that were directly apposed to the filamentous halo surrounding the ribbon body (Schnee et al., 2005; Obholzer et al., 2008). We observed a similar vesicle density along WT and *ribeye b-EGFP* ribbon perimeters (Fig. 1*B–D*,*I*; vesicle density per 500 nm, WT: 12.07 ± 0.80 vesicles*; ribeye b-EGFP*: 11.79 ± 0.76 vesicles, *p* = 0.80). The average size of individual vesicles was also similar at WT and *ribeye b-EGFP* ribbons (vesicle area, WT: 2236 nm^2^ ± 129.1*; ribeye b-EGFP*: 2345 ± 73.7 nm^2^, *p* = 0.46). In contrast, we found that synaptic vesicles were slightly closer to the ribbon in *ribeye b-EGFP* hair cells compared with WT (Fig. 1*B–D*,*L*; vesicle to ribbon distance, WT: 55.52 ± 3.65 nm*; ribeye b-EGFP*: 45.43 ± 2.99 nm, *p* = 0.039), which indicates that vesicle tethering might be slightly altered in the *ribeye b-EGFP* transgenic line. Importantly, our cross-section measurements revealed a significantly greater number of synaptic vesicles (1.3× more) associated with *ribeye b-EGFP* ribbons compared with WT ribbons (Fig. 1*J*; associated or tethered vesicles, WT: 17.67 ± 1.58 vesicles; *ribeye b-EGFP*: 22.53 ± 1.57 vesicles, *p* = 0.036). However, we did not observe a significantly greater number of vesicles docked at the active zones of enlarged ribbons, which we defined as vesicles beneath the ribbon and adjacent to the plasma membrane that were also opposed to the postsynaptic density (Fig. 1*B–D*,*K*; docked vesicles, WT: 2.83 ± 0.62; *ribeye b-EGFP*: 2.74 ± 0.34, *p* = 0.890). These results indicate that enlarged ribbons in *ribeye b-EGFP* transgenic hair cells have significantly more tethered vesicles but a similar number of docked synaptic vesicles compared with WT ribbons.

In addition to larger ribbons associated with postsynaptic densities (synaptic ribbons), we also observed ectopic ribbons in *ribeye b-EGFP* transgenic hair cells that were not associated with postsynaptic densities (Fig. 1*E–G*). Ectopic ribbons were rarely seen in WT TEM sections; we observed >60 ectopic ribbons in *ribeye b-EGFP* transgenic sections, but only 1 ectopic ribbon in a similar number of WT sections (*n* = 33 WT and *n* = 25 *ribeye b-EGFP* sections). Overall, we determined that most ectopic ribbons were located above the nucleus and were associated with membranous organelles or endosomes (Fig. 1*E–G*). Only a small subset of the ectopic ribbons were found along the membrane of hair cells (Fig. 1*E*), and the majority were associated with filaments in the cytosol (Fig. 1*F*) or near the apical cuticular plate of hair cells (Fig. 1*G*). Together, our TEM studies revealed that overexpression of Ribeye results in larger synaptic ribbons that are associated with more vesicles, as well as additional ectopic ribbons that populate the hair-cell body.

### Enlarged ribbons do not affect postsynapse morphology or synapse number

To assess whether ribbon enlargement also affected hair-cell postsynaptic morphology, we used immunohistochemistry to examine the afferent PSDs at ribbon synapses in lateral-line hair cells. We visualized afferent PSDs using an antibody against the PSD-95 family of MAGUKs and ribbons using antibodies specific to both paralogs of zebrafish Ribeye, Ribeye a and Ribeye b (Sheets et al., 2011). Because we observed numerous ectopic aggregates of Ribeye in our transgenic line (Figs. 1*A*,*E–G*, 2*B*,*D*), we quantified only Ribeye and MAGUK immunolabel at “complete” ribbon synapses (i.e., presynaptic Ribeye-labeled puncta juxtaposing MAGUK-labeled patches; Fig. 2*C*,*D*).

Because ribbon synapse components are too small to resolve accurately using conventional confocal microscopy, we approximated the sizes of presynaptic and postsynaptic components by examining the relative areas of Ribeye and MAGUK puncta. Corresponding to our TEM results, the relative areas of Ribeye puncta were significantly larger within *ribeye b-EGFP* hair cells compared with WT hair cells (Fig. 2*A–E*; ribbon area, normalized to WT median; WT: 1.082 ± 0.057 a.u., *n* = 192 ribbons; *ribeye b-EGFP*: 4.565 ± 0.252 a.u., *n* = 132 ribbons, *p* < 0.0001). By contrast, relative areas of MAGUK puncta were comparable with WT (Fig. 2*A–E*; PSD area, normalized to WT median; WT: 1.147 ± 0.059 a.u., *n* = 192 PSDs; *ribeye b-EGFP*: 1.311 ± 0.064 a.u., *n* = 210 PSDs, *p* = 0.052). We also examined whether the amount of PSD protein was altered at *ribeye b-EGFP* postsynapses by examining the integrated intensity of MAGUK immunolabel fluorescence per puncta, and found that, compared with *ribeye b-EGFP* puncta, amount of MAGUK within the PSDs was comparable with WT puncta (MAGUK integrated intensity, WT: 18,200 ± 1250 a.u., *n* = 192 PSDs; *ribeye b-EGFP*: 20,000 ± 1250 a.u., *n* = 210 PSDs, *p* = 0.1884). In addition, there was no significant difference in the number of intact ribbon synapses between *rib b-EGFP* and WT hair cells (Fig. 2*F*; synapses per hair cell, WT: 2.44 ± 0.10, *n* = 11 neuromasts; *ribeye b-EGFP*: 2.32 ± 0.13, *n* = 12 neuromasts, *p* = 0.468), and the PSDs appeared to correctly localize adjacent to the ribbons (Fig. 2*B*,*D*). These results indicate that enlarging the ribbon via overexpression of Ribeye b does not proportionally affect PSD size, nor does it change the number of ribbon synapses.

### Ca_V_1.3a channels are less tightly clustered at enlarged ribbon synapses

**Figure 3.**
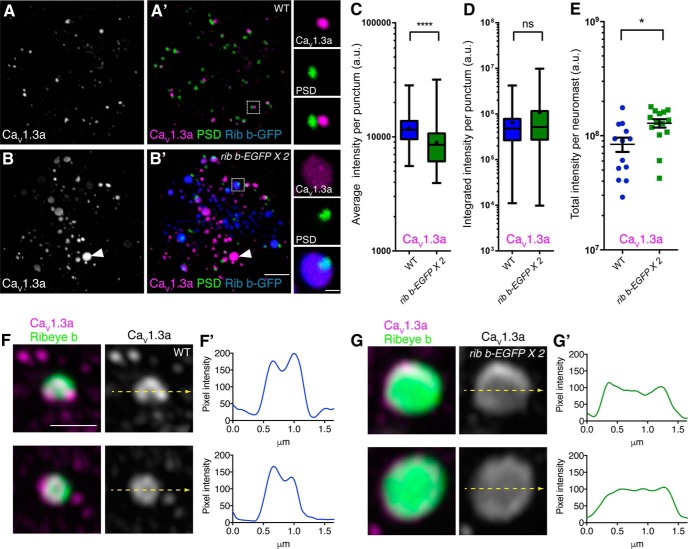
Ca_V_1.3a channel immunolabel is less clustered at enlarged ribbons. ***A***, ***B***, Immunolabel of Ca_V_1.3a in WT (***A***) and *ribeye b-EGFP* (***B***) hair cells at 5 dpf. ***A′***, ***B′***, Overlay of Ca_V_1.3a (magenta) and the PSD label MAGUK (green) in WT (***A′***) and *ribeye b-EGFP* (blue) (***B′***) hair cells. Right inset, Single ribbon; corresponds to boxed ROI in ***A′***, ***B′***. ***B***, ***B′***, White arrowheads indicate an enlarged ectopic ribbon with no PSD, yet relatively strong Ca_V_1.3a immunolabel. ***C***, ***D***, Ca_V_1.3a-immunolabeled puncta localized to complete synapses (i.e., adjacent to PSDs) were examined. ***C***, ***D***, The average Ca_V_1.3a immunolabel intensity at sites adjacent to PSD label is greater at WT synapses compared with *ribeye b-EGFP* synapses (***C***), whereas the total or integrated Ca_V_1.3a immunolabel intensity per punctum is unchanged between these groups (***D***) (*n* = 282 WT and *n* = 125 *ribeye b-EGFP* Ca_V_1.3a puncta in ***C***, ***D***). ***E***, The total amount of Ca_V_1.3a immunolabel per neuromast is greater in *ribeye b-EGFP* transgenics compared with WT. This includes Ca_V_1.3a immunolabel at both ectopic ribbons and at complete (PSD apposing) ribbon synapses (*n* = 13 WT and *n* = 14 *ribeye b-EGFP* neuromasts). ***F***, ***G***, Two example Airyscan images of Ca_V_1.3a and Ribeye b immunolabel at WT (***F***) and *ribeye b-EGFP* ribbons (***G***). ***F***, ***G***, Right, Ca_V_1.3a immunolabel alone. Left, Ca_V_1.3a (magenta) and Ribeye b (green) immunolabel merged. ***F′***, ***G′***, Intensity profile plots represent the pixel intensity of the yellow line drawn through the Ca_V_1.3a immunolabel in ***F***, ***G***. ***C***, ****p < 0.0001 (Mann–Whitney *U* test). ***E***, *p = 0.01 (unpaired *t* test). Scale bars: ***B′***, 5 μm (same scale bar for ***A***, ***B′***); **B**, inset, 1 μm; ***F***, 1 μm (same scale bar for ***F***, ***G***).

Previously, we reported that the voltage-gated calcium channel Ca_V_1.3, the presynaptic calcium channel in mammalian and zebrafish hair cells, is localized to synaptic ribbons (juxtaposing a PSD) and to ectopic aggregates of Ribeye b-EGFP (Fig. 3*B*,*B′*) (Sheets et al., 2011). First, we determined whether Ca_V_1.3a distribution was affected at enlarged synaptic ribbons. We reasoned that more tightly clustered Cav1.3a immunolabeled puncta would, on average, display higher average fluorescent intensities, and we therefore examined the average immunolabel intensity of Ca_V_1.3a puncta adjacent to a postsynapse. We found, at synapses with enlarged synaptic ribbons, the average intensity of Ca_V_1.3a immunolabel was reduced compared with WT synapses (Fig. 3*A–C*; Ca_V_1.3a average intensity, WT: 11,900 ± 205 a.u., *n* = 282 synapses; *ribeye b-EGFP*: 8930 ± 334 a.u., *n* = 125 synapses, *p* < 0.0001). Interestingly, the integrated immunolabel intensity per puncta, which represents the total amount of Ca_V_1.3a per synapse, was comparable between *ribeye b-EGFP* and WT synapses (Fig. 3*D*; Ca_V_1.3a integrated intensity, WT: 6.50 × 10^5^ ± 0.38 a.u., *n* = 282 synapses; *ribeye b-EGFP*: 1.08 × 10^6^ ± 0.14 a.u., *n* = 125 synapses, *p* = 0.156).

As our confocal imaging indicated that Ca_V_1.3a labeling was less clustered at *ribeye b-EGFP* synapses, we subsequently examined Ca_V_1.3a channel labeling more closely at synapses using super resolution microscopy. We observed that Ca_V_1.3a channels appeared less clustered at enlarged ribbons compared with WT ribbons (Fig. 3*F*,*G*). Profile plots of Ca_V_1.3a immunolabel showed multiple peaks at WT synapses (Fig. 3*F′*), corresponding with clustered Ca_V_1.3a labeling at the ribbon (Fig. 3*F*). In *ribeye b-EGFP* transgenic synapse, Ca_V_1.3a labeling appeared more diffuse and spread out over a larger area (Fig. 3*G*,*G′*). Collectively, these data suggest that there are a similar number of Ca_V_1.3a channels at enlarged synaptic ribbons compared with WT, but the channels cluster at a lower density.

Similar to what we previously observed, in *ribeye b-EGFP* hair cells, there were many additional ectopic ribbons with no associated PSD; these ectopic ribbons were also able to recruit Ca_V_1.3a channels (Sheets et al., 2011) (Fig. 3*B*,*B′*, example, arrowhead). When we summed the intensities of both ectopically and synaptically localized Ca_V_1.3a immunolabel, we observed significantly more overall Ca_V_1.3a present in *ribeye b-EGFP* transgenic hair cells compared with WT hair cells (Fig. 3*E*; total Ca_V_1.3a intensity per neuromast, WT: 3.29 × 10^5^ ± 0.47 a.u., *n* = 13 neuromasts; *ribeye b-EGFP*: 5.03 × 10^5^ ± 0.42 a.u., *n* = 14 neuromasts, *p* = 0.010). These findings confirm that ectopic ribbons can recruit Ca_V_1.3a without a postsynapse and suggest that the amount of Ca_V_1.3a allocated to each synaptic ribbon is fixed. Perhaps, if the amount of Ca_V_1.3a at synaptic ribbons is fixed, then increases in Ribeye and ribbon size can influence Ca_V_1.3a channel distribution and consequently serve as a mechanism to adjust the overall density of Ca_V_1.3a channels at the active zone.

### Whole-cell calcium currents, but not capacitance measurements, are increased in hair cells with enlarged ribbons

To investigate how enlarged ribbons altered hair-cell function, we performed whole-cell recordings from lateral-line hair cells of WT and of *ribeye b-EGFP* transgenic larvae. The resting membrane potential of Ribeye b-EGFP-expressing hair cells was similar to that measured in WT hair cells (*V*_m_, WT: −69.5 ± 4.8 mV, 3–5 dpf, *n* = 4; *ribeye b-EGFP*: −72.8 ± 3.8 mV, 3–5 dpf, *n* = 6, *p* = 0.602). In addition, at rest, we were unable to detect any difference in cell membrane capacitance between genotypes (WT: 2.8 ± 0.3 pF, *n* = 25; *ribeye b-EGFP*: 3.3 ± 0.6 pF, *n* = 31, *p* = 0.491), which suggests that the overall size of the hair cells is similar. Furthermore, there were no differences in the complement of K^+^ currents, which included *I*_K,Ca_ and *I*_A_ as previously described, between WT and *ribeye b-EGFP* hair cells (data not shown) (Olt et al., 2014, 2016b).

**Figure 4.**
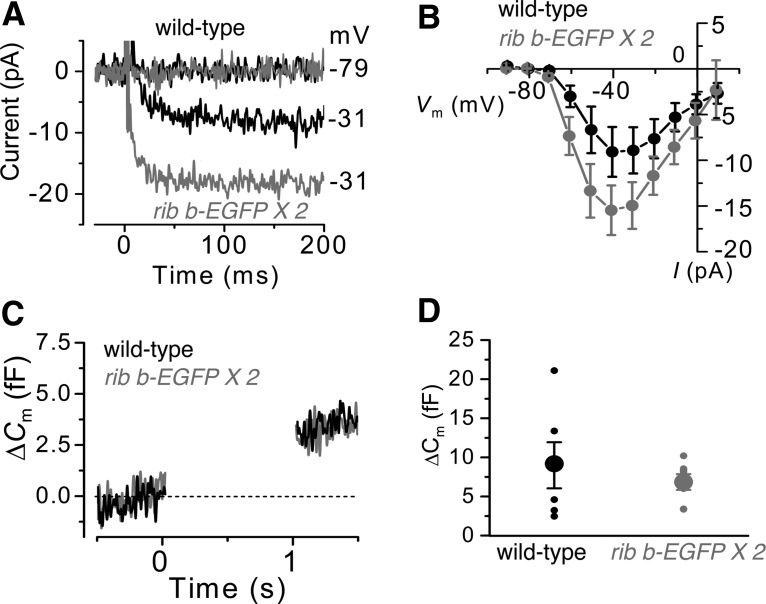
Calcium currents and capacitance measurements in hair cells with enlarged ribbons. ***A***, Calcium currents (*I*_Ca_) recorded from WT (7 dpf, black) or *ribeye b-EGFP* hair cells (5 dpf, gray). Currents were elicited by a series of depolarizing voltage steps in 10 mV increments (200 ms in duration) from the holding potential of −79 mV. For clarity, only the trace at the holding potential and at the peak of *I*_Ca_ are shown. ***B***, *I–V* curves of whole-cell *I*_Ca_ in WT (black) and *ribeye b-EGFP* (gray) hair cells (5–8 dpf) (*n* = 13 WT and *n* = 10 *ribeye b-EGFP*). At the peak of *I*_Ca_ (−31 mV), calcium currents were significantly larger in *ribeye b-EGFP* hair cells (*p* > 0.05). ***C***, Changes in membrane capacitance (ΔC_m_) recorded from hair cells of WT (5 dpf, black) and *ribeye b-EGFP* transgenics (6 dpf, gray). Recordings were obtained in response to 1.0 s voltage steps from the holding potential of −79 mV to near the peak of *I*_Ca_ (−31 mV). ***D***, Average ΔC_m_ elicited following 1.0 s depolarization step to −31 mV from hair cells of both WT (6–8 dpf, black) and *ribeye b-EGFP* (5–8 dpf, gray) hair cells was not significantly different using a *t* test (*n* = 6 WT and *n* = 7 *ribeye b-EGFP*).

Our immunolabel results indicate that the distribution of Ca_V_1.3a channels at *ribeye b-EGFP* synapses is disrupted compared with WT synapses (Fig. 3). We therefore examined whether the calcium currents (*I*_Ca_) expressed in *ribeye b-EGFP* hair cells were altered relative to WT hair cells (Fig. 4*A*,*B*). *I*_Ca_ was recorded using 2.8 mm extracellular calcium at 28.5°C, and in the presence of the K^+^ currents blockers 4-aminopyridine and tetraethyl ammonium in the Cs-based intracellular solution (see Materials and Methods; Fig. 4*A*, example *I_Ca_*). *Ribeye b-EGFP* hair cells had larger calcium currents compared with WT hair cells (Fig. 4*B*; at −31 mV, the peak of *I*_Ca_, WT: −7.0 ± 1.4 pA, *n* = 13; *ribeye b-EGFP*: −13.2 ± 1.8 pA, *n* = 10, *p* = 0.012).

In hair cells, voltage-gated calcium currents are tightly associated with vesicle fusion (i.e., exocytosis). To investigate whether *ribeye b-EGFP* hair cells had altered exocytosis, we measured changes in cell-membrane capacitance (Δ*C*_m_) following a 1.0 s depolarizing voltage step (Fig. 4*C*, example Δ*C*_m_) (Moser and Beutner, 2000; Johnson et al., 2009; Olt et al., 2014, 2016a). Despite the larger *I*_Ca_ in *ribeye b-EGFP* hair cells, the Δ*C*_m_ was similar to that measured in WT cells (Fig. 4*C*,*D*; Δ*C*_m_ 1.0 s step, WT: 9.0 ± 3.0 fF, *n* = 6; *ribeye b-EGFP*: 6.8 ± 1.0 fF, *n* = 7, *p* = 0.47), and similar to that reported in WT hair cells using the same recording conditions (Olt et al., 2014, 2016b). These data show that, despite more associated vesicles at enlarged ribbons and larger calcium currents in *ribeye b-EGFP* hair cells, the overall amount of exocytosis does not correspondingly increase in response to a 1.0 s stimulus.

### Calcium imaging confirms larger cytosolic calcium responses in hair cells with enlarged ribbons

**Figure 5.**
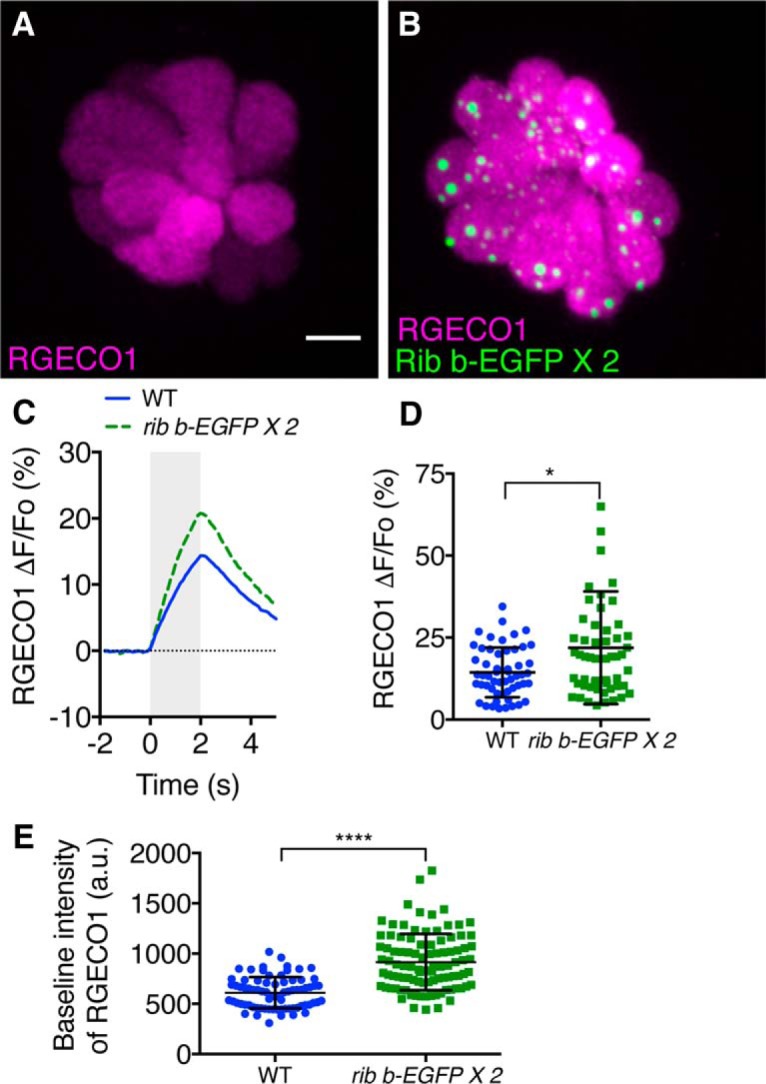
Mechanically evoked and baseline cytosolic calcium measurements are increased in hair cells with enlarged ribbons. ***A***, ***B***, Live, confocal image of hair cells expressing the calcium indicator RGECO1 (***A***) or RGECO1 and Ribeye b-EGFP (***B***). ***C***, Average mechanically evoked calcium response of hair cells expressing RGECO1 (WT, solid blue) or RGECO1 and Ribeye b-EGFP (*ribeye b-EGFP*, dashed green) at 5–6 dpf (*n* = 54 WT and *n* = 55 *ribeye b-EGFP* hair cells). Gray bar represents the duration of the mechanical stimulus (2.0 s). ***D***, Scatterplot of the magnitude of individual hair cell responses plotted in ***C***. ***E***, Baseline RGECO calcium signals in WT and *ribeye b-EGFP* transgenic hair cells (*n* = 74 WT and *n* = 96 *ribeye b-EGFP* hair cells). A Mann–Whitney *U* test was used to compare calcium responses and baseline calcium signals in ***D***, ***E***. *p < 0.05. ****p < 0.0001. Scale bar: ***A***, 5 μm.

To further test calcium dynamics in hair cells with enlarged ribbons, we examined mechanically evoked calcium responses in a transgenic line expressing RGECO1 in hair cells (Zhao et al., 2011; Maeda et al., 2014). RGECO1 is a red-shifted calcium indicator that enabled us to spectrally separate Ribeye b-EGFP and RGECO1 and examine calcium responses within the cytosol of hair cells with enlarged ribbons (Fig. 5*B*; RGECO1 plus 2 copies of *ribeye b-EGFP*) and WT ribbons (Fig. 5*A*; RGECO1 alone). After a 2.0 s stepwise deflection of the cupula of neuromasts, we measured and quantified mechanically evoked calcium responses (fluorescence response, ΔF/F_0_), in both genotypes. On average, calcium responses were significantly larger in hair cells with enlarged ribbons compared with WT hair cells (Fig. 5*C*,*D*; RGECO1 ΔF/F_0_, WT: 14.39 ± 1.03%, *n* = 54 hair cells; *ribeye b-EGFP*: 21.89 ± 2.32%, *n* = 55 hair cells, *p* = 0.02). This finding is consistent with what we observed in our whole-cell calcium current measurements, where hair cells with enlarged ribbons had significantly larger calcium currents. In addition to increased mechanically evoked calcium responses, we also observed that baseline calcium levels, as measured by baseline RGECO1 intensity, were significantly elevated in *ribeye b-EGFP* transgenic hair cells compared with WT hair cells (Fig. 5*E*; RGECO1 baseline, WT: 610.9 ± 18.01 a.u., *n* = 74 hair cells; *ribeye b-EGFP;* 916.0 ± 28.62 a.u., *n* = 96 hair cells, *p* < 0.0001). Overall, elevated baseline calcium and increased cytosolic calcium responses support the results from our whole-cell recordings and indicate that hair cells with enlarged ribbons have increased calcium signaling.

### Local calcium signals at ribbons are larger in hair cells with enlarged ribbons

**Figure 6.**
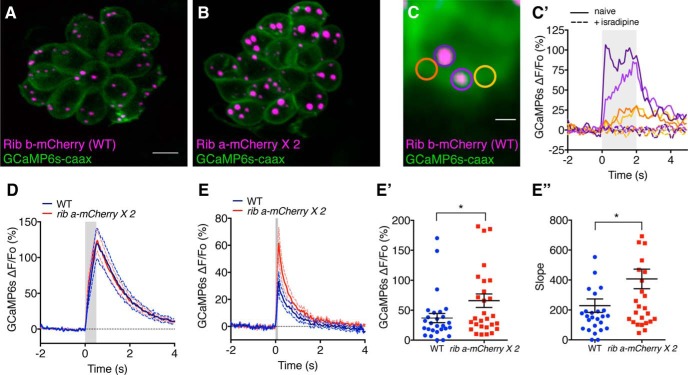
Ribbon-localized calcium signals are increased at enlarged ribbons. ***A***, ***B***, Live images of hair cells with WT-sized ribbons, *ribeye b-mCherry;GCaMP6s-CAAX* (***A***) and enlarged ribbons, *ribeye a-mCherry* × *2;GCaMP6s-CAAX* (***B***). ***C***, ***C′***, Two ribbons in a single hair cell are depicted in ***C***. In response to a saturating, 2.0 s, 5 Hz stimulus, in control hair cells (*ribeye b-mCherry*), focal calcium hotspots are observed at individual ribbons (purple ROIs) compared with an adjacent region (orange ROIs). Isradipine completely blocks all presynaptic calcium signals (***C′***, dashed traces). ***D***, ***E***, Average ribbon-localized calcium responses during a 0.5 s (***D***) or 0.1 s (***E***) stimulus at WT ribbons and enlarged ribbons. ***E′***, ***E″***, Data from ***E*** quantified with respect to magnitude (***E′***) and slope (***E″***). Measurements were performed at 4–5 dpf. ***D***, Traces represent the average response of *n* = 29 WT (*ribeye b-mCherry*) and *n* = 37 enlarged (*ribeye a-mCherry*) ribbons. ***E–E″***, Traces and data represent the average response of *n* = 27 WT (*ribeye b-mCherry*) and *n* = 29 enlarged (*ribeye a-mCherry*) ribbons. ***C′***, ***D***, ***E***, Gray bar represents the duration of the mechanical stimulus. ***D***, ***E***, Dashed lines indicate SEM. *p < 0.05 (Mann–Whitney *U* test). Scale bars: ***A***, 5 μm; ***C***, 1 μm.

Our immunohistochemistry data indicate that there are the same number of Ca_V_1.3a channels at enlarged ribbons compared with WT ribbons, but the channels cluster at a lower density. Functionally, our whole-cell recordings and cytosolic calcium imaging results show larger global calcium responses in *ribeye b-EGFP* hair cells. To determine whether reduced Ca_V_1.3a channel clustering altered the local calcium responses at enlarged synaptic ribbons, we established a method to detect ribbon-localized calcium responses at ribbons. To do this, we used a transgenic zebrafish that expresses the calcium indicator GCaMP6s (Chen et al., 2013) in hair cells, localized to the plasma membrane with a CAAX motif (Fig. 6*A–C*) (Jiang et al., 2017). We predicted that this line could be used to detect presynaptic calcium signals adjacent to ribbons. To test this prediction, we created an additional transgenic line, *ribeye b-mCherry*, which has WT-sized ribbons (see Materials and Methods) to mark ribbon location to measure GCaMP6s-CAAX, ribbon-localized calcium signals. Using this double-transgenic line (Fig. 6*A*), we were able to measure robust, mechanically evoked calcium responses (fluorescence response, ΔF/F_0_) basally in hair cells, in response to deflection of the cupula of neuromasts (Fig. 6*C*,*C′*). The presynaptic calcium signals were localized within a focal hotspot located at ribbons (Fig. 6*C*,*C′*). We detected ribbon-localized calcium signals by centering an ROI with a 1 μm diameter over an individual ribbon (Fig. 6*C*). Compared with an adjacent ROI, calcium signals at the ribbon were greater (peak GCaMP6s calcium response for a 2.0 s stimulus, on ribbon: 158.10 ± 0.23%; off ribbon: 32.08 ± 0.04%, *n* = 12 hair cells, *p* < 0.0001, examples shown in Fig. 6*C*,*C′*). All presynaptic signals were blocked below detection by application of the L-type calcium channel antagonist isradipine, with complete block observed in 12 of 12 cells in response to a saturating stimulus (Fig. 6*C′*, example dashed traces). Together, our imaging and pharmacological results support the use of GCaMP6s-CAAX for detecting local, Ca_V_1.3a-dependent calcium signals at ribbons.

Because of spectral overlap between EGFP and GCaMP6s, the *ribeye b-EGFP* and *GCaMP6s-CAAX* transgenic lines are not compatible for imaging. To overcome this obstacle, we created an additional transgenic line with high levels of Ribeye expression. Instead of EGFP, we fused Ribeye to mCherry. Similar to the *ribeye b-EGFP* transgenic line, when in-crossed, *ribeye a-mCherry* transgenic ribbons were enlarged compared with WT ribbons (see Materials and Methods; Fig. 6*B*). This enlargement is not observed in our control *ribeye b-mCherry* transgenic line, where ribbon size is comparable with WT (see Materials and Methods; Fig. 6*A*). We used these two mCherry lines to mark ribbon position and determine whether ribbon-localized calcium signals (using *GCaMP6s-CAAX*) were altered at enlarged ribbons (*ribeye a-mCherry* × *2*) compared with control ribbons (*ribeye b-mCherry*). We found, during a 0.5 s step stimulus, that the magnitude of the ribbon-localized calcium responses at enlarged ribbons was similar compared with control (Fig. 6*D*). We reasoned that the GCaMP6s-CAAX indicator could become saturated during this stimulus. Therefore, we also measured the magnitude of the ribbon-localized calcium response during a shorter, 0.1 s step stimulus. During a 0.1 s step, we observed that calcium responses were larger at enlarged ribbons compared with control ribbons (Fig. 6*E*,*E′*; peak GCaMP6s ribbon calcium response, *ribeye b-mCherry* [WT]: 36.94 ± 7.69%, *n* = 27 ribbons; *ribeye a-mCherry* [enlarged]: 65.89 ± 11.27%, *n* = 29 ribbons, *p* = 0.042). In addition, for the 0.1 s step stimulus, the slope (rate of change toward peak) of the response was increased at enlarged ribbons compared with control (Fig. 6*E″*; slope, *ribeye b-mCherry* [WT]: 228.8 ± 45.04, *n* = 27 ribbons; *ribeye a-mCherry* [enlarged]: 407.2 ± 65.41, *n* = 29 ribbons, *p* = 0.041).

In addition to synaptic ribbons, we also examined GCaMP6s-CAAX responses at ectopic ribbons. We observed that, despite associating with Ca_V_1.3a channels (Fig. 3*B*,*B′*), at ectopic ribbons, we were not able to measure a GCaMP6s-CAAX signal that was substantially different from the surrounding background (*n* = 15 ectopic ribbons). This suggests that ectopic ribbons may not contribute to the differences in global calcium signals that we measured using whole-cell recordings or the calcium indicator RGECO1.

Overall, our measurements indicate that, for short stimuli, ribbon-localized calcium responses at enlarged synaptic ribbons are both faster and larger compared with responses at WT-sized synaptic ribbons. An increase in calcium signals at enlarged ribbons is in line with our measurements of whole-cell calcium currents and our experiments using cytosolic RGECO1 where we observed increased calcium responses. Together, these results indicate that both local ribbon-localized calcium dynamics as well as global calcium dynamics are increased with ribbon enlargement.

### Ribbon enlargement alters spontaneous and evoked afferent activity

Presynaptic measurements of capacitance changes to estimate exocytosis were unable to detect any differences in evoked neurotransmission between hair cells from WT or *ribeye b-EGFP* transgenic fish. Because of the limited number of ribbons and associated vesicles in zebrafish hair cells, it is likely that a large depolarizing stimulus may be required to detect changes in exocytosis using capacitance measurements. This could confound our ability to detect subtle differences in vesicle fusion between genotypes. Therefore, we examined whether postsynaptic activity was affected in *ribeye b-EGFP* transgenic fish by performing extracellular recordings of action currents from the cell bodies of the afferent neurons that innervate lateral-line hair cells (Trapani and Nicolson, 2010). For these recordings, both spontaneous and evoked action currents (spikes) are generated by neurotransmitter release from hair cells that is dependent on Ca_V_1.3a activity (Trapani and Nicolson, 2011; Olt et al., 2016b). Spontaneous spikes result from a receptor potential that is within the activation range of Ca_V_1.3a channels when the hair cell is not stimulated. Evoked spikes occur when hair cells are stimulated and result from depolarizing mechanotransduction currents that lead to rapid activation of Ca_V_1.3a channels (Moser and Beutner, 2000; Jørgensen and Kroese, 2005; Trapani and Nicolson, 2011).

**Figure 7.**
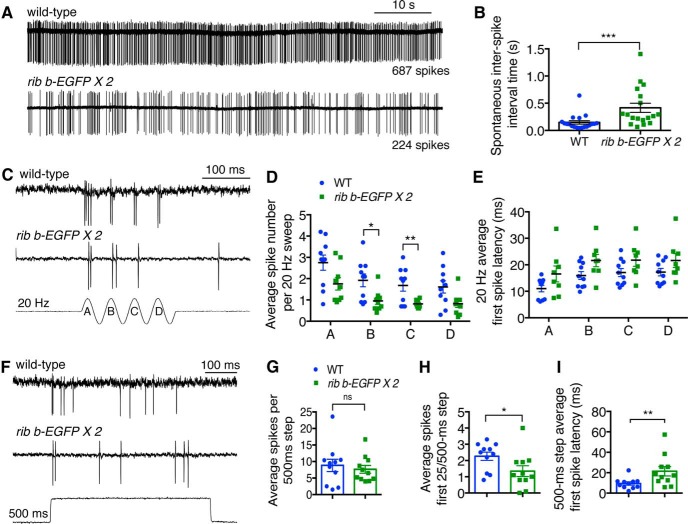
Enlarged ribbons disrupt evoked and spontaneous afferent activity. ***A***, Representative example of a 60 s recording of spontaneous action currents from WT and *ribeye b-EGFP* afferent neurons. ***B***, Quantification of mean spontaneous interspike intervals from WT and *ribeye b-EGFP* (*n* = 19 WT and *n* = 18 *ribeye b-EGFP* neurons). ***C***, Example trace from a WT and *ribeye b-EGFP* afferent neuron in response to a 200 ms 20 Hz sine-wave stimulus. Each stimulus has four cycles with positive (stimulatory) Phases A–D. ***D***, Average spike number for each stimulus Phase (A–D) is reduced in *ribeye b-EGFP* compared with WT (*n* = 8 WT and *n* = 10 *ribeye b-EGFP* neurons). ***E***, Mean time to first spike for each stimulus phase in *ribeye b-EGFP* compared with WT. ***F***, Example sweep from the same WT and *ribeye b-EGFP* afferent neuron as ***C***, in response to a 500 ms step stimulus. ***G***, Average spike number per 500 ms step is the same for *ribeye b-EGFP* compared with WT (*n* = 11 neurons for WT and *ribeye b-EGFP*). ***H***, Spike number for the first 25 ms of the 500 ms step stimulus is significantly lower in in *ribeye b-EGFP* compared with WT. ***I***, The mean first spike latency is longer in *ribeye b-EGFP* neurons compared with WT. ***D***, ***E***, ***G***, ***H***, ***I***, *p < 0.05 (unpaired *t* test). **p < 0.01 (unpaired *t* test). ***p < 0.001 (unpaired *t* test). ***D***, ***E***, Multiple *t* tests were corrected for multiple comparisons using the Holm–Sidak method. ***B***, Mann–Whitney *U* test was used.

Using our whole-animal, *in vivo* approach, we examined spontaneous and evoked activity from afferent neurons of WT and *ribeye b-EGFP* transgenic fish. We first examined the spontaneous activity of single afferent neurons and found that hair cells with enlarged ribbons displayed significantly reduced rates of spontaneous activity with corresponding larger interspike intervals compared with WT hair cells (Fig. 7*A*,*B*; spontaneous interspike interval, WT: 0.147 ± 0.031 s, *n* = 19 cells; *ribeye b-EGFP*: 0.416 ± 0.085 s, *n* = 18 cells, *p* = 0.0006). This result suggests that, despite having more synaptic vesicles localized to larger ribbons compared with WT ribbons, *ribeye b-EGFP* transgenic hair cells have a lower probability of vesicle fusion at rest.

We next examined evoked afferent activity to determine whether ribbon size played a role in the encoding of mechanical stimuli. Stimuli were presented as two different types of neuromast cupula deflections: a 200 ms sine-wave at 20 Hz and a 500 ms square-step stimulus. During a 20 Hz sine-wave stimulus, we observed fewer spikes during two phases of the stimulus that activated hair cells in *ribeye b-EGFP* larvae compared with WT larvae (Fig. 7*C*,*D*). In contrast, during a 500 ms step stimulus, we were unable to detect a difference in the average number of spikes in *ribeye b-EGFP* afferents compared with WT afferents (Fig. 7*F*,*G*; spikes per 500 ms step, WT: 8.84 ± 1.86 spikes, *n* = 11 cells; *ribeye b-EGFP*: 7.64 ± 1.19 spikes, *n* = 11 cells, *p* = 0.67). These results are consistent with our capacitance measurements where we observed no difference between *ribeye b-EGFP* transgenic and WT hair cells after 1.0 s step stimuli (Fig. 4*C*,*D*). When reconciling why the number of spikes was reduced for 20 Hz, but not the 500 ms step stimulus, we noted that there were fewer spikes during the first 25 ms of the 500 ms step in *ribeye b-EGFP* transgenic fish compared with WT (Fig. 7*H*; WT: 2.26 ± 0.26 spikes, *n* = 11 cells; *ribeye b-EGFP*: 1.35 ± 0.60 spikes, *n* = 11 cells, *p* = 0.022). This initial reduction of spikes suggests that fewer vesicles fuse at enlarged ribbons during the onset of a stimulus. For the 20 Hz sine-wave stimulus, the depolarizing phase of each 50 ms cycle (Fig. 7*C*, labeled A-D for the 200 ms stimulus duration) is 25 ms, which may not be enough time for enlarged ribbons to achieve WT levels of exocytosis. Together, the decrease in afferent activity in response to short-phasic 20 Hz stimuli and at the onset of a longer-lasting saturating stimulus supports the hypothesis that there is reduced exocytosis at enlarged ribbons at stimulus onset.

Our examination of encoding of stimulus onset also revealed that the mean of the first spike latency in response to a 500 ms step was longer in *ribeye b-EGFP* afferent neurons compared with WT neurons (Fig. 7*I*; first spike latency for a 500 ms step, WT: 9.47 ± 1.59 ms, *n* = 11 cells; *ribeye b-EGFP*: 23.40 ± 6.27 ms, *n* = 11 cells, *p* = 0.007). In addition, first spike latencies appeared slightly longer for each phase of the 20 Hz sine-wave stimulus; however, these individual values were not statistically different from WT (Fig. 7*E*). Overall, hair cells with enlarged ribbons resulted in a significantly reduced rate of spontaneous activity in downstream afferent neurons, and an alteration in evoked activity with fewer postsynaptic spikes and longer first spike latencies at stimulus onset.

## Discussion

A specific role for the synaptic ribbon in sensory encoding has been remarkably challenging to define. Genetic studies aimed to perturb ribbons have yet to give a complete story of what features enable ribbon synapses to encode a particular auditory or vestibular stimulus. We used a transgenic zebrafish line that overexpresses Ribeye and enlarges hair-cell ribbons to examine how ribbon size alters the morphology and function of hair-cell synapses. Morphologically, hair cells with enlarged ribbons had more associated vesicles and failed to tightly cluster presynaptic Ca_V_1.3a channels, whereas PSDs appeared unaltered. Functionally, whole-cell calcium currents, cytosolic calcium responses, and ribbon-localized calcium responses were larger in hair cells with enlarged ribbons. Despite increased calcium signaling, enlarging ribbons resulted in reduced afferent spontaneous activity and disruptions in evoked release at the onset of stimuli. Overall, our results suggest that alterations to presynaptic ribbon size can influence postsynaptic activity and the encoding properties of ribbon synapses.

### Ribeye and Ca_V_1.3 channel clustering

Numerous reports have shown that Ca_V_1.3 channels are clustered beneath hair-cell ribbons and are required for fast exocytosis at ribbon synapses (Roberts, 1994; Moser and Beutner, 2000; Frank et al., 2010; Sheets et al., 2012; Wong et al., 2014). Two recent studies examining the genetic reduction of Ribeye in zebrafish hair cells and mouse retina have also indicated a close relationship between Ribeye levels and Ca_V_1 localization. In these studies, disruption of Ribeye levels resulted in mislocalization of Ca_V_1.3a and Ca_V_1.4, respectively (Lv et al., 2016; Maxeiner et al., 2016). We also observed a strong association between Ca_V_1.3a and Ribeye; Ca_V_1.3a was recruited to all ribbons, including ectopic ribbons generated by overexpression of Ribeye (Fig. 3*B*,*B′*). Yet our observations in this study reveal that augmenting Ribeye at synapses is not sufficient to recruit additional Ca_V_1.3a channels to synapses. We observed that the number of Ca_V_1.3a at individual synaptic ribbons did not scale up with increased Ribeye levels and ribbon enlargement. Instead, the integrated intensity of Ca_V_1.3a channel immunolabel at enlarged synaptic ribbons was similar to WT (Fig. 3*D*) but spread out over larger ribbon areas (Fig. 3*A–D*,*F*,*G*). This result suggests that Ca_V_1-channel density at the synapse may be regulated independently of Ribeye, perhaps through another ribbon-associated scaffolding protein, such as Bassoon, or a RIM protein (Frank et al., 2010; Jung et al., 2015).

### The ribbon's influence on coupling calcium influx and vesicle fusion

Previous work compiled from TEM studies has shown that larger ribbons have more ribbon-associated vesicles (Nouvian et al., 2006; Matthews and Fuchs, 2010; Graydon et al., 2011). Likewise, our TEM data also indicate that enlarged ribbons have an increased number of tethered vesicles compared with WT ribbons, which predicts the potential for an increase in vesicle fusion (Fig. 1*J*). In addition, our measurements of whole-cell calcium currents and ribbon-localized calcium signals found that calcium responses were elevated in hair cells with enlarged ribbons (Figs. 4*A*,*B*, 6*E*,*E′*). Yet despite larger calcium currents and more tethered vesicles at enlarged ribbons, we observed no increase in evoked activity and a significant reduction in spontaneous vesicle fusion (Fig. 7). These results are similar to what has been observed in mammalian auditory IHCs, where larger ribbons are correlated with elevated calcium currents and less spontaneous activity (Taberner and Liberman, 2005; Liberman et al., 2011).

Our results suggest that other presynaptic factors may be important to couple calcium influx and vesicle fusion. There are several scenarios wherein ribbon enlargement could impact this coupling and ultimately the probability of vesicle fusion driving both spontaneous and evoked activity. These mechanisms include changes in Ca_V_1.3 channel activation properties, an inhibitory effect on vesicle release by the ribbon itself, and/or a disruption of coupling between presynaptic calcium signaling and vesicle fusion.

Alterations to Ca_V_1.3 channel activation threshold could result in decreased spontaneous release and set a higher threshold for fusion of the vesicles at stimulus onset (Brandt et al., 2005; Magistretti et al., 2015). Recent work in mouse IHCs found that, compared with smaller ribbons, Ca_V_1.3 channels at larger ribbons within the same hair cell were activated at more depolarized membrane potentials creating a higher threshold for activation (Ohn et al., 2016). By contrast, we saw a similar activation of the calcium current in transgenic hair cells compared with WT hair cells (Fig. 4*B*). Additionally, we did not observe differences in the resting potential in our whole-cell recordings (see Results). Overall, our data support that the reduced afferent activity we observed in our transgenic is not likely due to altered Ca_V_1.3a channel activation.

Another possibility is the physical size or composition of the ribbon could impact vesicle fusion. For example, previous studies in bipolar cells suggest one function of the ribbon may be to stabilize a group of vesicles for evoked release while minimizing their spontaneous release (Zenisek, 2008; Vaithianathan et al., 2013). We did observe that overexpression of Ribeye decreased the distance between tethered vesicles and the ribbon (Fig. 1*L*), which provides some evidence that Ribeye may play a role in vesicle tethering, and perhaps could act to stabilize vesicles at the ribbon. If enlarged ribbons restrain vesicles and inhibit their release, this mechanism could explain why we observed larger calcium currents (Fig. 4*A*,*B*) and more tethered vesicles (Fig. 1*J*) in hair cells with enlarged ribbons, but less spontaneous afferent activity and disruptions in evoked activity (Fig. 7).

In addition to the physical size of the ribbon and the amount of Ribeye influencing vesicle fusion, reduced Ca_V_1.3a channel clustering observed at enlarged ribbons (Fig. 3*F*,*G*) could impact the coupling of presynaptic calcium influx to vesicle fusion. Coupling of presynaptic calcium influx to vesicle release in hair cells has been previously described by two models. The first is the nanodomain model, in which the opening of one or a few Ca_V_1.3 channels is coupled to exocytotic calcium sensors on adjacent vesicles that trigger fusion (Brandt et al., 2005; Goutman and Glowatzki, 2007; Wong et al., 2014). In the second, the microdomain model, the opening of several, more distant Ca_V_1.3 channels is necessary to collectively raise calcium levels to overcome mobile calcium buffering and drive vesicle fusion (Roberts, 1994; Beaumont et al., 2005). Our current study was not designed to study the spread of calcium at the presynapse; therefore, our results cannot be used to conclusively distinguish between either of these models. Regardless of the mechanism, it is possible that reduced Ca_V_1.3 channel clustering impacts the coupling of presynaptic calcium influx with vesicle fusion. In future studies, it will useful to design experiments using this transgenic line to directly explore how enlarged ribbons affect the coupling of presynaptic calcium influx and vesicle fusion in more detail.

### The relationship between ribbon size and afferent neuron sensitivity

Ribbon size correlates with the sensitivity of auditory nerve fibers innervating IHCs of the cochlea, with smaller ribbons associated with low-threshold/high-spontaneous rate fibers and larger ribbons associated with high-threshold/low-spontaneous rate fibers (Taberner and Liberman, 2005; Liberman et al., 2011). A recent study characterizing the firing properties of rat auditory neurons supports that differences observed in afferent spike timing can be attributed to the properties of presynaptic vesicle release mechanisms (Wu et al., 2016). When we enlarged synaptic ribbons in the zebrafish lateral line, we observed a shift in afferent fiber sensitivity (i.e., enlarged ribbons resulted in a reduced rate of spontaneous firing in afferent neurons; Fig. 7*A*,*B*). This finding is consistent with the correlation described in IHCs, suggesting that varying ribbon size may be a sufficient mechanism for tuning afferent sensitivity.

In addition to reduced spontaneous spike rates in afferent neurons, enlarged ribbons also resulted in an increase in first spike latency at stimulus onset. Moreover, we observed decreases in evoked spikes in response to shorter-duration stimuli (20 Hz, 25-ms stimulus per phase), and during the onset (first 25 ms) of longer, sustained stimuli. In larval zebrafish, the fidelity and latency of the first spike are an important fast encoding mechanism used to rapidly generate an escape reflex that can be critical for survival (Troconis et al., 2017). In mammals, the first spike is important for perceptual encoding (Heil, 2004; Johansson and Birznieks, 2004; Chase and Young, 2007) as well as sound localization (Furukawa and Middlebrooks, 2001).

Our observations in the zebrafish lateral line reveal that enlargement of ribbons in hair cells is sufficient to alter afferent activity. This work provides insight into the mechanisms of hair-cell synapse heterogeneity and suggests that hair cells could use a simple strategy, varying ribbon size, to achieve sensitivity over a broad dynamic range of stimuli. It is possible that our study is relevant from a clinical perspective, as recent work has found that ribbon enlargement following moderate noise exposure is accompanied by coding deficits in auditory nerve fibers (Song et al., 2016). It will be useful in future studies to determine whether morphological changes to hair-cell ribbons directly contribute to functional deficits associated with noise exposure.
